# Wheeze Recognition Algorithm for Remote Medical Care Device in Children: Validation Study

**DOI:** 10.2196/28865

**Published:** 2021-06-17

**Authors:** Chizu Habukawa, Naoto Ohgami, Takahiko Arai, Haruyuki Makata, Morimitsu Tomikawa, Tokihiko Fujino, Tetsuharu Manabe, Yoshihito Ogihara, Kiyotaka Ohtani, Kenichiro Shirao, Kazuko Sugai, Kei Asai, Tetsuya Sato, Katsumi Murakami

**Affiliations:** 1 Department of Pediatrics Minami Wakayama Medical Center Tanabe Japan; 2 Omron Healthcare Co, Ltd Muko Japan; 3 Arai Pediatric Clinic Yamagata Japan; 4 Makata Pediatrics & Allergy Clinic Yamaguchi Japan; 5 Odasaga Pediatrics and Allergy Sagamihara Japan; 6 Kokura Allergy Clinic Kokura Japan; 7 Manabe Pediatric Clinic Ebina Japan; 8 Ogihara Kids Clinic Zama Japan; 9 Kirin Kids Allergy Clinic Sagamihara Japan; 10 Shirao Clinic of Pediatrics and Pediatric Allergy Hiroshima Japan; 11 Sugai Children's Clinic Pediatrics/Allergy Hiroshima Japan; 12 Department of Psychosomatic Medicine Sakai Sakibana Hospital Sakai Japan

**Keywords:** asthma, children, infant, wheezing, wheeze recognition algorithm, pediatrics, remote, medical devices, validation, home management, algorithm, detection, chronic illness

## Abstract

**Background:**

Since 2020, peoples’ lifestyles have been largely changed due to the COVID-19 pandemic worldwide. In the medical field, although many patients prefer remote medical care, this prevents the physician from examining the patient directly; thus, it is important for patients to accurately convey their condition to the physician. Accordingly, remote medical care should be implemented and adaptable home medical devices are required. However, only a few highly accurate home medical devices are available for automatic wheeze detection as an exacerbation sign.

**Objective:**

We developed a new handy home medical device with an automatic wheeze recognition algorithm, which is available for clinical use in noisy environments such as a pediatric consultation room or at home. Moreover, the examination time is only 30 seconds, since young children cannot endure a long examination time without crying or moving. The aim of this study was to validate the developed automatic wheeze recognition algorithm as a clinical medical device in children at different institutions.

**Methods:**

A total of 374 children aged 4-107 months in pediatric consultation rooms of 10 institutions were enrolled in this study. All participants aged ≥6 years were diagnosed with bronchial asthma and patients ≤5 years had reported at least three episodes of wheezes. Wheezes were detected by auscultation with a stethoscope and recorded for 30 seconds using the wheeze recognition algorithm device (HWZ-1000T) developed based on wheeze characteristics following the Computerized Respiratory Sound Analysis guideline, where the dominant frequency and duration of a wheeze were >100 Hz and >100 ms, respectively. Files containing recorded lung sounds were assessed by each specialist physician and divided into two groups: 177 designated as “wheeze” files and 197 as “no-wheeze” files. Wheeze recognitions were compared between specialist physicians who recorded lung sounds and those recorded using the wheeze recognition algorithm. We calculated the sensitivity, specificity, positive predictive value, and negative predictive value for all recorded sound files, and evaluated the influence of age and sex on the wheeze detection sensitivity.

**Results:**

Detection of wheezes was not influenced by age and sex. In all files, wheezes were differentiated from noise using the wheeze recognition algorithm. The sensitivity, specificity, positive predictive value, and negative predictive value of the wheeze recognition algorithm were 96.6%, 98.5%, 98.3%, and 97.0%, respectively. Wheezes were automatically detected, and heartbeat sounds, voices, and crying were automatically identified as no-wheeze sounds by the wheeze recognition algorithm.

**Conclusions:**

The wheeze recognition algorithm was verified to identify wheezing with high accuracy; therefore, it might be useful in the practical implementation of asthma management at home. Only a few home medical devices are available for automatic wheeze detection. The wheeze recognition algorithm was verified to identify wheezing with high accuracy and will be useful for wheezing management at home and in remote medical care.

## Introduction

Since 2020, people’s lifestyle worldwide has been largely changed due to the COVID-19 pandemic. In the medical field, many patients are afraid to become infected with the virus in clinics and prefer remote medical care. In remote medical care, the physician cannot examine the patient directly, and therefore it is important for the patients to accurately convey their condition to the physician. Thus, remote medical care should be implemented, and adaptable home medical devices are required for this purpose.

Wheeze is the most important exacerbation sign in various respiratory diseases among all age groups [1-3]. Bronchial asthma is one of the typical diseases that requires home management, in which physicians detect wheezes by auscultation as acute exacerbation. Therefore, for the home management of asthma, caregivers should be aware of wheezing in small children at night and adolescents during play exercise. Moreover, physicians are mostly dependent on reports from family members and caregivers regarding symptoms, who may have different judgment criteria for wheezes [4-6]. Therefore, a high-accuracy objective method to detect wheezes would be beneficial for physicians and patients’ families or caregivers. To the best of our knowledge, no appropriate home device has been used to detect wheezing as a mild exacerbation sign to date.

Computerized lung sound analysis, especially computerized wheeze detection, is a more objective and standardized method, which can overcome limitations of subjective auscultation [3,7]. In the medical field, technical innovation has engendered telemedicine and home-based therapy; however, the practical use of these technologies has been limited. For respiratory diseases, lung sounds represent simple physical data, which have no value by themselves and are only clinically important when evaluated with identical criteria of judgment by a physician [8-10].

For remote medical care, we developed a new handy home medical device with automatic wheeze recognition algorithms, which is available for clinical use in noisy environments such as a pediatric consultation room or at home. Moreover, the examination time is only 30 seconds because small children cannot endure long examination times without crying or moving [11]. In this study, we aimed to validate the automatic wheeze recognition algorithm based on wheeze sound characteristics with this new small handy device for clinical use in young children, including infants, at different institutions.

## Methods

### Participants

Ten institutions that have pediatric respiratory and allergy specialists were registered for this study. All participants were outpatient children attending the entry clinic and hospital located in Japan (Yamagata, Kanagawa, Hiroshima, Yamaguchi, Fukuoka, and Wakayama) between September 24, 2019 and November 22, 2019. All participants were brought into the hospital for the treatment of recurrent wheezes with cough and dyspnea. Written informed consent was obtained from all participants or their legal guardians. The study protocol was approved by the ethics committee of Minami Wakayama Medical Center [approval number 2016-22(5)]. All participants aged ≥6 years were diagnosed with bronchial asthma, and their asthmatic severities were classified as mild asthma according to the 2017 Japanese Pediatric Guideline for the Treatment and Management of Asthma [12]. The children were treated with a leukotriene receptor antagonist and/or inhaled corticosteroid in accordance with the guidelines [12]. Participants aged ≤5 years had reported at least three episodes of wheezes and had been treated with a leukotriene receptor antagonist or without medicine for long-term management.

### Study Procedures

A specialist physician examined all participants using a stethoscope and simultaneously recorded lung sounds during tidal breathing in the pediatric consultation room for at least 30 seconds. Recordings were obtained from the upper right anterior chest region at the second intercostal space in the midclavicular line of the chest wall. Recorded lung sounds (with or without wheezes) were then listened to by the same specialist physician who recorded lung sounds, and then confirmed and classified the sounds in accordance with previous methods [11].

A total of 177 recordings were designated as “wheeze” files and 197 were designated as “no-wheeze” files. In addition, each specialist physician who recorded lung sounds differentiated wheezes from lung sound samples, including inspiratory and expiratory lung sounds, nasal congestion, crying, and voices.

### Sound Recording and Analysis

#### HWZ-1000T Device

**Figure 1:**
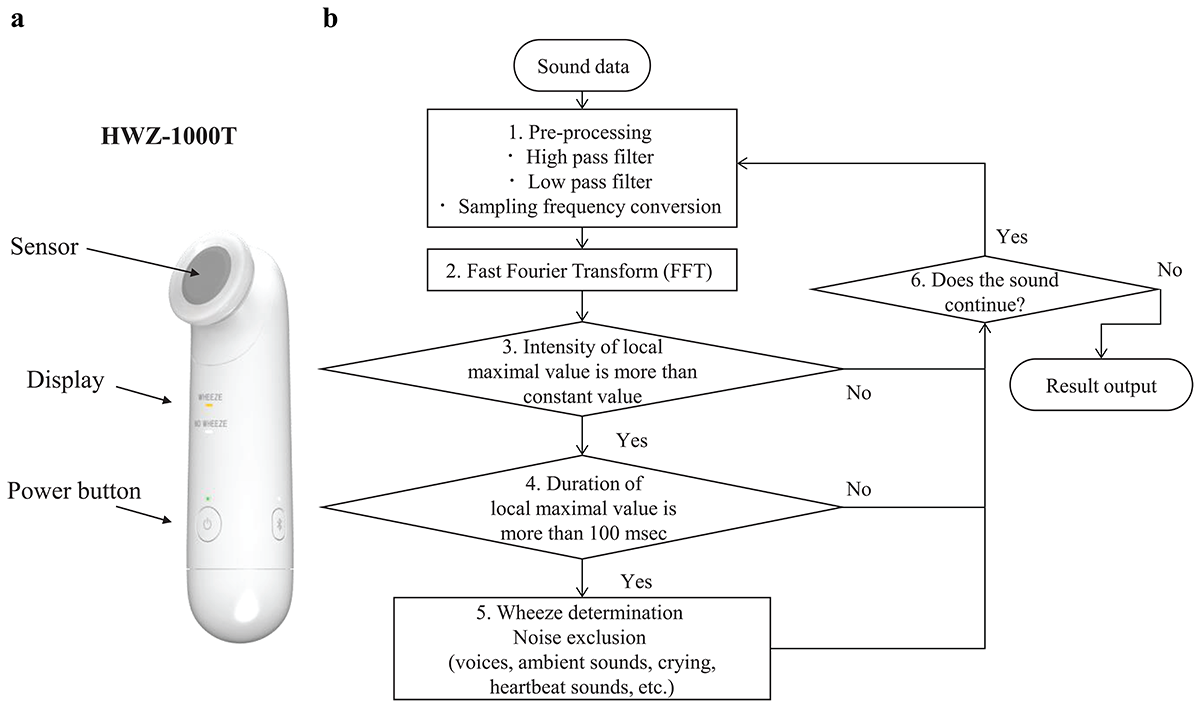
Sound recording device and flowchart of the wheeze detection algorithm. FFT: fast Fourier transform.

Lung sounds were recorded using a small handy device with an automatic wheeze recognition algorithm (HWZ-1000T, Omron Healthcare Corporation, Kyoto, Japan) (Figure 1a).

Two microphones are installed in the sensor unit, one for recording lung sounds and the other for recording environmental sounds. The microphone for recording lung sounds makes the judgment of wheezes through skin contact. Recorded lung sounds are processed using a wheezing recognition algorithm implemented in the internal central processing unit to automatically determine the presence or absence of wheezing, and then the results of wheezing judgment can be displayed on the device after 30 seconds. To analyze recorded wheeze sounds and compare judgment results by physicians who recorded wheezing with the automatic wheeze recognition algorithm, we attached a micro-SD memory card to the HWZ-1000 T device for confirmation of recorded lung sounds. The outline of the algorithm is described below.

#### Characteristics of Wheeze by Lung Sounds Analysis

According to the Computerized Respiratory Sound Analysis guidelines, the dominant frequency and duration of a wheeze were set to >100 Hz and >100 ms, respectively [13]. Furthermore, a previous report described the frequency range of a typical wheeze to be between 100 and 5000 Hz [1]. The maximum duration of a wheeze is within the expiratory duration. A wheeze detection algorithm was developed based on this definition.

**Figure 2:**
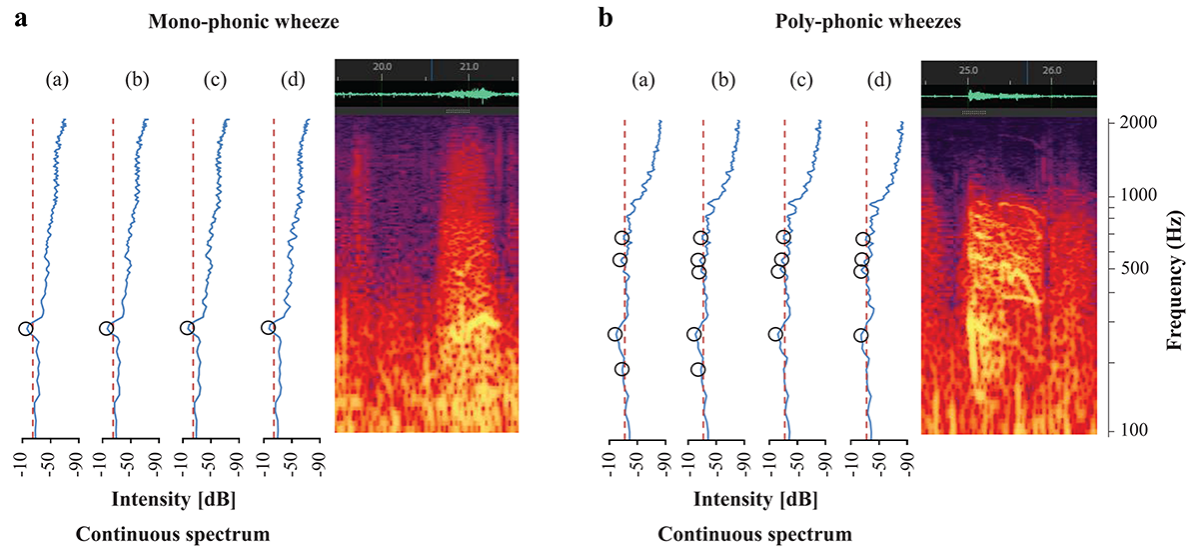
Monophonic and polyphonic wheezes. A: (a) Spectrum 1: Fast Fourier transform (FFT) frame, 21.000-21.372 s; (b) Spectrum 2: FFT frame, 21.018-21.390 s; (c) Spectrum 3: FFT frame, 21.036-21.408 s; (d) Spectrum 4: FFT frame, 21.054-21.426 s. B: (a) Spectrum 1: FFT frame, 25.000-25.372 s; (b) Spectrum 2: FFT frame, 25.018-25.390 s; (c) Spectrum 3: FFT frame, 25.036-25.408 s; (d) Spectrum 4: FFT frame, 25.054-25.426.

Figure 2 (right panels) shows a typical wheeze spectrogram, with time (seconds) and frequency (Hz) on the horizontal and vertical axes, respectively. The sound intensity (dB) is shown as color and brightness. A continuous wheeze spectrum was created based on the lung sound analysis. On the left panels, horizontal axes show intensity (dB) and the vertical axis shows frequency (Hz). Wheeze sounds are shown as horizontal bars with intensity corresponding to peaks in the power spectrum display [1,13-15]. Wheeze sounds were classified into two types. On the left panel, a wheeze shows only one peak with intensity as a monophonic wheeze, and on the right panel, a wheeze shows many peaks with intensity as polyphonic wheezes. Wheezing is considered monophonic when only one pitch is heard, whereas it is considered polyphonic when multiple frequencies are simultaneously perceived. Polyphonic wheezing indicates more severe bronchial constriction than monophonic wheezing [3,16,17].

### Wheeze Recognition Algorithm

Based on the definition of wheeze characteristics, a flowchart was created for the developed wheeze recognition algorithm from the sound collection to automatically detect wheezes to generate results. Details of the wheeze recognition algorithm were provided in our previous report [11] (Figure 1b). We describe the wheeze recognition algorithm with the following overall approach that consisted of five phases.

In step 1, sound data were preprocessed using high and low bandpass filters. Data were resampled at a sampling rate of 11.025 kHz and at a 16-bit quantization rate.

In step 2, fast Fourier transform (FFT), the most well-known acoustic analysis method, was used. FFT analyzes the intensity for each frequency of sound data. The sound data were preprocessed using a hamming window of 4096 points (372 ms), and processing was repeated every 128-point (18 ms) increase in the sound data [18-20].

Since the lung sound spectra had many local maximum points each time, in step 3, some local maximum points higher than the threshold were extracted as candidates for wheeze sounds. Black-circled points indicate the extracted local maximum points. The orange dotted line represents the threshold value used to determine the local maximum point. Threshold values were determined from overall sound pressure levels between 90 and 5000 Hz.

In step 4, whether the local maximum points selected in step 3 continued for >100 ms was determined according the definition of wheeze characteristics [13]. Continuous local maximum values selected in step 4 still included wheeze sounds and other noises, including voices, ambient sounds, crying, and heartbeat sounds.

In step 5, threshold values were determined using feature values to eliminate noises. To finally determine the presence of wheezes using both lung and ambient sounds, feature values of wheezing candidates selected in step 4 were calculated. Finally, if at least one wheeze sound was heard in a file, it was identified as a wheeze file, whereas if no wheeze sound was heard in a file, it was identified as a no-wheeze file.

For validation, we compared the judgment of wheeze sound recognition using the algorithm to assess all files that were discriminated by each specialist physician who recorded lung sounds.

### Statistical Analysis

The results fell into one of the following four categories: actual positives that were correctly predicted as positives (true positives, TP); actual positives that were wrongly predicted as negatives (false negatives, FN); actual negatives that were correctly predicted as negatives (true negatives, TN); and actual negatives that were wrongly predicted as positives (false positives, FP). We analyzed the sensitivity (TP/TP+FN), specificity (TN/TN+FP), positive predictive value (PPV= TP/TP+FP), and negative predictive value (NPV= TN/TN+FN) using the wheeze recognition algorithm results in all data files [21,22]. PPV is defined as the probability that files identified as “wheeze” files by the specialists were also identified as “wheeze” files by the algorithm. NPV is the probability that files identified as “no-wheeze” files by the specialists were also identified as “no-wheeze” files by the algorithm.

Statistical analysis was performed using R software version 3.4.1. Patient characteristics are presented as the mean and range. Wheeze sound characteristics are presented as mean (SD) and range. Noise ratios in each sound discriminated by the algorithm are presented as a percentage of all noises. The relationship between age and sensitivity of wheeze recognition was analyzed using the Jonckheere-Terpstra test [23,24]. A *P* value <.05 was considered statistically significant.

## Results

### Participant Characteristics

Table 1 shows the participant characteristics.

**Table 1:** Participant characteristics (N=374).

Characteristic			Value
Age (months), mean (SD)			44.3 (31.6)
**Age category (months), n (%)**			
	4-11	54 (14.4)	
	12-23	70 (18.7)	
	24-35	52 (13.9)	
	36-47	38 (10.2)	
	48-59	48 (12.8)	
	60-71	19 (5.1)	
	72-83	18 (4.8)	
	84-95	45 (12.0)	
	96-107	30 (8.0)	
**Sex, n (%)**			
	Male	241 (64.4)	
	Female	133 (35.6)	
Height (cm), mean (SD), range			96.2 (28.5), 56.0-133.0
Weight (kg), mean (SD), range			15.6 (6.8), 4.5-34.0

### Classification of Recorded Sounds and Wheeze Characteristics

Table 2 shows the classification of recorded sounds in all lung sound samples and wheeze sound characteristics. If the wheeze contained essentially a single frequency, it was classified as a monophonic wheeze, whereas it was classified as a polyphonic wheeze if it contained several frequencies [25].

**Table 2:** Classification of sounds in all recorded sound files (N=1201).

Sound classification		Value
**Characteristics of wheeze sounds, mean (SD), range**		
	Frequency (Hz)	321 (178), 100-1600
	Intensity (dB)	21.2 (7.0), 5.0-45.0
	Duration (ms)	331 (220), 100-2538
**Type of wheeze sounds, n (%)**		
	Monophonic wheeze	457 (38.1)
	Polyphonic wheeze	744 (61.9)
	Total	1201
**Noise, n (%)**		
	Nasal congestion	108 (19.4)
	Physician’s voice	155 (27.8)
	Ambient crying or voice	294 (52.8)
	Total	557

### Number of Local Maximum Points of Wheeze Sounds

Table 3 shows the number of local maximum points of wheeze sounds, for a total of 1201 in all recorded sounds. Among these, 457 (38.1%) wheezes were found to have one local maximum point. In addition, 352 (29.3%) wheezes had two local maximum points. Overall, <3 local maximum points accounted for >67.4% of all wheeze sounds.

**Table 3:** Number of local maximum points of wheeze sounds in all recorded sounds (N=1201).

Number of local maximum points	Wheeze sounds, n (%)
1	457 (38.1)
2	352 (29.3)
3	187 (15.6)
4	104 (8.7)
5	58 (4.8)
6	18 (1.5)
7	16 (1.3)
8	3 (0.2)
9	5 (0.4)
10	1 (0.1)

### Accuracy of Wheeze Recognition

Table 4 displays the wheeze recognition results using the wheeze detection algorithm. The sensitivity, specificity, PPV, and NPV for wheeze recognition in all data files were 96.6% (171/177), 98.5% (194/197), 98.3% (171/174), and 97.0% (194/200), respectively.

**Table 4:** Results per file obtained using the newly developed wheeze recognition algorithm for children.

Identification by the algorithm	Specialist’s diagnosis by stethoscope	
	Wheeze sound	No-wheeze sound
Wheeze sound	TP^a^=171	FP^b^=3
No-wheeze sound	FN^c^=6	TN^d^=194

^a^TP: true positive.

^b^FP: false positive.

^c^FN: false negative.

^d^TN: true negative.

### Influence of Age and Sex on the Sensitivity of Wheeze Detection

The sensitivity and specificity of wheeze detection are shown in Table 5. The sensitivity and specificity of wheeze detection were not influenced by age and sex.

**Table 5:** Influence of age and sex on the sensitivity of wheeze detection.

Group		Wheeze data			No-wheeze data		
		N	TP^a^	Sensitivity (%) (95% CI)	N	TN^b^	Specificity (%) (95% CI)
**Age (months)**							
	0	24	23	95.8 (78.9-99.9)	30	28	93.3 (77.9-99.2)
	1	45	43	95.6 (84.9-99.5)	25	25	100 (86.3-100)
	2	27	26	96.3 (81.0-99.9)	25	25	100 (86.3-100)
	3–8	81	79	97.5 (91.4-99.7)	117	116	99.1 (95.3-100)
**Sex**							
	Male	110	106	96.4 (89.3-97.6)	131	130	99.2 (92.2-98.6)
	Female	67	65	97.0 (90.3-99.3)	66	64	97.0 (88.6-98.3)
Total		177	171	96.6 (92.8-98.7)	197	194	98.5 (95.6-99.7)

^a^TP: true positive.

^b^TN: true negative.

### Automatic Differentiation of Wheezes From Other Sounds Using the Wheeze Detection Algorithm

**Figure 3:**
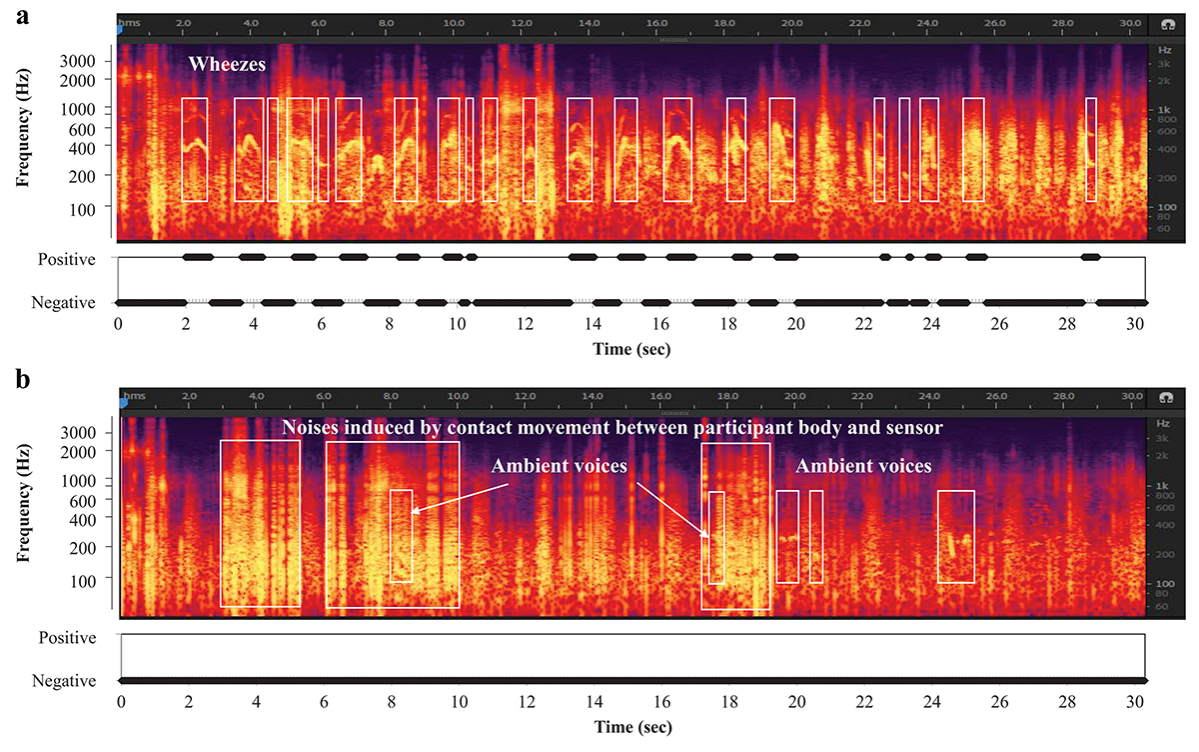
Results of wheeze recognition with wheezing before and after inhalation.

Figure 3a shows the wheeze recognition results with wheezing before inhalation. Wheezes (white squares) were accurately detected by the automatic recognition algorithm. Figure 3b shows the no-wheeze results after inhalation, including crying and voices (arrows). The other noises were effectively discriminated from wheeze sounds; wheezes were automatically detected, whereas heartbeat sounds, voices, and crying were automatically identified as no-wheeze sounds by the wheeze recognition algorithm.

## Discussion

Wheezes in children, including infants, were successfully detected using the newly developed small handy device with a wheeze recognition algorithm. This algorithm could precisely discriminate wheezes from other noises in an environment with various sounds. Furthermore, based on wheeze characteristics, the automatic wheeze recognition algorithm could detect even mild wheezes in crying infants recorded for 30 seconds in a pediatric consultation room. Therefore, we have successfully developed a real-time wheeze detection system with higher robustness for clinical use.

Studies on automated wheeze detection have been performed for various clinical conditions [17,18]. These detection systems have been developed in the past three decades. In 1995, Gavriely [13] published the details of a technological approach for automated digital data acquisition and breathing sound processing. This commercial device, PulmoTrack, enabled automated and continuous wheeze monitoring. Boner et al [20] reported that monitoring wheezes during sleep was useful when treating children with asthma, and that the duration of wheezes during the recording was correlated with peak expiratory flow rate changes. Therefore, automated wheeze detection may be useful for the management of children with wheezes, especially infants. A meta-analysis found that computerized lung sound analysis had relatively high sensitivity and specificity in a small number of studies [26,27]. Although wheeze detection systems have been successfully implemented, they have not been used clinically in children (including infants), owing to several problems that can be encountered while using automated wheeze detection systems [28-30].

One important factor is the varied wheeze intensities among children. Wheezes are continuous adventitious lung sounds that are superimposed on breath sounds. According to new definitions in the current Computerized Respiratory Sound Analysis guidelines, the dominant frequency of a wheeze is usually >100 Hz with a duration of >100 ms [25]. The most common features of detecting wheezes are the use of different wheeze peak shapes in the time-frequency plane, such as amplitude spectrum, continuity, spread, sparseness, and kurtosis. Continuous local maximum points of intensity in the spectra, which are considered as the most common features associated with wheezes during lung analysis, were analyzed. Wheeze spectra and spectrograms have many local maximum points when using FFT. Other technologies such as PulmoTrack provide respiratory rates, inspiratory/expiratory time ratios, wheeze rate during the recording duration, and wheeze duration. PulmoTrack detected >3 local maximum points. However, <3 local maximum points accounted for >67.4% of all wheeze sounds in this study and in our previous study, and our wheeze recognition algorithm could detect >1 local maximum point. Moreover, our algorithm could also detect mild wheezes [11].

Prodhan et al [31] used PulmoTrack in a pediatric intensive care unit and reported that wheeze detection was more accurate compared with that performed by hospital staff. Nurses’ judgment of wheezing has been reported to differ from that of physicians and caregivers by nearly 60% [4]. The judgment of wheezing may also differ among each physician, which could be due to the variety of wheezing sounds and many local maximum points from a few weak local maximum points. In this verification study, although 10 specialists who recoded wheezing performed independent assessments, we succeeded in obtaining highly accurate results of wheezing judgment. In other words, our developed wheeze recognition algorithm can accurately detect weak and mild wheezing, which may be judged differently by specialists. Consequently, our algorithm exhibited higher sensitivity over other wheeze detection technologies.

Another problem to be overcome is that a short examination time is required to accurately detect wheezes, and a simple procedure should be clinically used in small children. In small children, including infants, recording lung sounds without crying, moving, or being distracted by the attached adhesive pads or belt is difficult. Therefore, we selected a method that can record within a 30-second period by attaching a microphone to the chest wall by hand. In a previous study on 214 children, including 30 infants, the sensitivity of wheeze detection using our algorithm was not affected by age [12]. Moreover, this study comprising 374 children, including 54 infants, showed that the sensitivity of wheeze detection using our algorithm was not affected by age or sex. In addition, the small handy device is useful size for children and their caregivers.

A highly precise noise-canceling technology should be developed for clinical use for young children. Recording lung sounds in a noisy clinic requires more rigorous postprocessing than recording in a quiet room to compensate for the noise present in the acoustic signal. Therefore, the efficiency of classification algorithms may differ. These inconsistencies would lead to difficulties in interpreting and translating study outcomes, and they have hindered the clinical use of computerized lung sound analysis devices, especially in children [25]. To improve the accuracy of the algorithm for automatically detecting wheezing, various methods have been developed with the aim of eliminating the influence of human voices and various environmental sounds, but they have not been put into practical use [32-34].

Algorithms such as neural networks, vector quantization, Gaussian mixture model classification systems, and support vector machines have been used to analyze spectral features. A support vector machine is a supervised machine-learning algorithm used for both classification and regression [28,29]. The presence of wheezes can be identified using a decision tree with classifiers of other noises. The decision tree is a method that can classify sounds according to detailed differences in sound features. Heartbeat sounds typically last for <100 ms. Voices and other sounds produce noises of higher decibel levels on the environmental microphone than wheezes on the lung sound microphone. Crying is louder on the lung sound microphone than on the environment microphone, but shows different continuous pattern ranges compared with wheeze sounds. Therefore, no-wheeze sounds could be automatically distinguished from wheezes using the wheeze detection algorithm. We discriminated wheezes from environmental noise based on different wheeze sound characteristics. Thus, no-wheeze sounds could be automatically distinguished from other noises in a noisy pediatric consultation room.

This study has a few limitations. First, the use of the algorithm at home should have been validated. Second, in case of severe airway obstruction, it did not demonstrate any audible lung sounds (known as “silent chest”); however, patients with a severe condition show a pale face or difficulty breathing. Therefore, caregivers can easily recognize these as exacerbation signs.

Wheezing often occurs in the absence of a doctor, such as during the night, at home, or during exercise, and possibly even in the absence of a parent. Our new home medical device, equipped with a highly accurate algorithm that is not affected by environmental noise, can easily detect wheezing and may be able to properly detect asthma attacks at home in the absence of a doctor, which will further be useful for remote medical care.

We successfully developed a real-time wheeze detection system with higher robustness for clinical application using lung sound analysis in children and infants. We successfully discriminated wheezes from other noises such as heartbeats, voices, and crying using the wheeze detection algorithm in a noisy pediatric consultation room. This practical implementation may provide beneficial information for physicians and parents of children and infants. In the future, we plan to verify whether use of this device can be expanded to include older children and adults. We hope to use the novel home medical device equipped with this algorithm, which could help improve the safety of children with asthma and respiratory illnesses.

## Abbreviations

FFT: fast Fourier transform
FN: false negative
FP: false positive
NPV: negative predictive value
PPV: positive predictive value
TN: true negative
TP: true positive
